# Positive Association of Plasma Trimethylamine-N-Oxide and Atherosclerosis in Patient with Acute Coronary Syndrome

**DOI:** 10.1155/2022/2484018

**Published:** 2022-11-03

**Authors:** Wanwen Kong, Junyi Ma, Ying Lin, Weiyu Chen

**Affiliations:** ^1^Department of Cardiology, Shunde Hospital, Guangzhou University of Traditional Chinese Medicine, China; ^2^Guangzhou University of Traditional Chinese Medicine, China; ^3^The Heart Research Institute, The University of Sydney, Australia

## Abstract

**Aim:**

Atherosclerosis is the major cause of acute coronary syndrome (ACS) which is a significant contributor to both morbidity and mortality in the world. The microbiome-derived metabolite trimethylamine-N-oxide (TMAO) has aroused great interest and controversy as a risk factor of atherosclerosis. Therefore, in this study, we aimed at investigating whether plasma TMAO can be a risk factor of atherosclerosis in coronary artery of patients with ACS and how this relates to lipids and proinflammatory cytokines in plasma.

**Methods:**

We enrolled consecutive patients with ACS who underwent percutaneous coronary intervention (PCI). Gensini scoring was used to evaluate angiographic atherosclerosis in the coronary artery of the patients. 13 patients were divided into low (Gensini score < 25), 33 into intermediate (Gensini score 25-50), and 81 into severe atherosclerosis (Gensini score ≥50). Plasma TMAO, vasculitis factors, and cardiovascular biomarkers were measured by clinical biochemistry, intima-media thickness (IMT) of carotid artery was determined by the Color Doppler ultrasound, and the atherosclerotic lesion in coronary artery was assessed in PCI.

**Results:**

Plasma TMAO concentrations were positively associated with Gensini score (OR = 0.629, *p* < 0.001) and Gensini subgroup (R = 0.604, *p* < 0.001). Plasma TMAO concentrations in patients with severe coronary atherosclerosis were higher than those of patients with moderate coronary atherosclerosis, and the plasma TMAO concentrations of patients with moderate coronary atherosclerosis were higher than those of patients with mild coronary atherosclerosis, the difference was statistically significant [4.73 (3.13, 4.62) versus 1.13 (0.63, 3.34) versus 0.79 (0.20, 1.29), *p* < 0.001], respectively. Furthermore, ROC analysis showed that plasma TMAO could identify the severity of atherosclerosis (*p* < 0.001). The AUC of TMAO for severe atherosclerosis was 0.852 (95%CI = 0.779 − 0.925). The sensitivity and specificity of TMAO for identifying severe atherosclerosis are 96.3% and 63.0% when the cut-off value of TMAO was set at 1.2715 pg/ml. Furthermore, logistic regression analysis showed plasma TMAO concentrations were positively associated with severity of atherosclerosis in coronary artery (OR = 1.934, 95%CI = 1.522 − 2.459, *p* < 0.001). For all that, negatively association was observed between TMAO and age (OR = −0.224, *p* < 0.05), B-type natriuretic peptide (BNP) (OR = −0.175, *p* < 0.05), and interleukin-8 (IL-8) (OR = −0.324, *p* < 0.001), while positive association was observed between TMAO and nitric oxide (NO) (OR = 0.234, *p* < 0.01). However, there is no obvious association was observed between Gensini score and cardiovascular biomarkers, vasculitis factors, and carotid IMT, respectively.

**Conclusion:**

Our cross-sectional observation suggested that plasma TMAO concentrations positively associated with coronary atherosclerosis in ACS patients and serve as a risk factor for severe atherosclerosis. Plasma TMAO also correlated with age, BNP, IL-8, and NO. However, no obvious association was found between atherosclerosis with vasculitis factors and cardiovascular biomarkers in this study, and there was no conclusive evidence showing TMAO enhance atherosclerosis via regulation of inflammation or lipid.

## 1. Introduction

Acute coronary syndromes (ACS) are acute ischemic syndromes characterized by fresh thrombosis caused by the rupture or erosion of coronary atherosclerotic plaques. ACS is an acute and critical disease of coronary heart disease (CHD). ACS includes acute ST elevation myocardial infarction (STEMI), non-ST elevation myocardial infarction (NSTEMI), and unstable angina (UA). Atherosclerosis is the major cause of CHD [1]. Smoking, hypertension, diabetes, hyperlipidemia, inflammation, and other risk factors have shown to increase the risk of atherosclerosis [2]. And clinical studies have shown that treatment specific to reduce these risk factors, such as quitting smoking, lowering blood pressure, lowering blood sugar, lowering blood lipids, and anti-inflammatory intervention, can alleviate atherosclerosis. However, with treatment targeting to these risk factors, patients with ACS after PCI still have a high mortality rate [3]. With standard treatment and management of those risk factors, the incidence of adverse cardiovascular events in patients with acute myocardial infarction is still as high as about 7% [4]. Therefore, there may be other risk factors which contribute to the process and development of atherosclerosis in coronary artery. Identifying these unknown risk factors of atherosclerosis is significantly important to provide therapeutic guidelines for coronary atherosclerosis, thereby reducing incidence, morbidity, and mortality of CHD.

Recent studies have shown that intestinal flora and its metabolites affect human health and the occurrence of cardiovascular diseases, e.g., CHD, heart failure, hypertension, and atrial fibrillation [5]. When the intestinal flora metabolizes choline and lecithin, it produces trimethylamine (TMA). After TMA is absorbed into the blood, it is catalyzed and oxidized by liver flavin monooxygenase (FMO) to produce a metabolite called trimethylamine-oxide (TMAO). TMAO was significantly correlated with CHD. Tang et al., conducted a 3-year prospective cohort study with 4007 participants and reported that participants with high plasma TMAO concentrations had higher incidence rate of atherosclerosis than those with low plasma TMAO concentrations [6]. The mechanism of TMAO promoting atherosclerosis may be through affecting lipid metabolism and endothelial function [7]. Consistent with the underlying mechanism proposed by Tang et al., other clinical studies have shown that TMAO enhances cholesterol loading in macrophages and promotes the formation of atherosclerotic plaque via regulation on cholesterol metabolism [8]. TMAO can also elevate the expression of atherogenic receptors (differentiation cluster 36 and scavenger receptor A) and increase the risk of atherosclerotic plaque formation [9]. TMAO is also reported to promote the release of a range of inflammatory factors, e.g., interleukin-8 (IL-8) and endothelin-1 (ET-1), which can lead to vascular endothelial damage and resulting atherosclerosis [10].

Therefore, in addition to the traditional CHD risk factors, TMAO may play a role as a potential risk factor in the development of coronary atherosclerotic plaques as well as CHD. However, the relationship between TMAO and ACS by coronary angiography has not been established. In this study, we measured plasma concentrations of TMAO, analyzed the cross-sectional relationship between TMAO and ACS risk by coronary angiography, and reported a positively association between plasma TMAO concentrations and atherosclerosis in coronary artery of ACS.

### 1.1. Study Design and Population

This cross-sectional study was based on the case data obtained from the patients with ACS recruited in the Shunde Hospital of Guangzhou University of Traditional Chinese Medicine. The study was approved by the Research Ethics Committee of Shunde Hospital of Guangzhou University of Traditional Chinese Medicine (Protocol: KY-2020113). In brief, this cross-sectional study is aimed at identifying the risk factors of patients with ACS after PCI by evaluation and analysis of the corresponding clinical outcomes. This cross-sectional study included patients with ACS who underwent PCI at Shunde Hospital of Guangzhou University of Traditional Chinese Medicine from May 2021 to March 2022. Blood and plasma samples, biochemical data, clinical characteristics, coronary angiography, demographic data, electrocardiogram data, risk factors, and PCI procedures were collected and recorded in lab archives managed by the Shunde Hospital. A total of 144 patients with ACS underwent PCI were recruited initially. 17 were excluded due to the Gensini score data not being available or presence of acute infection, acute renal failure, or hepatic failure. Eventually, 127 patients were included in this study, with 94 males and 33 females. There were 40 diabetic and 87 nondiabetic patients.

### 1.2. The Diagnosis of Acute Coronary Syndrome

According to the guidelines for Rapid Emergency Diagnosis and Treatment of Acute Coronary Syndrome (2019): (1) ST-elevation myocardial infarction (STEMI); cTn>99th upper normal reference value (ULN) or CK-MB>99th. In the ULN, the echocardiography (ECG) has an elevated ST arch back, with one or more of the following conditions: persistent ischemic chest pain, ECG showing abnormal segmental ventricular wall motion, and abnormal coronary angiography. (2) non-ST-elevation myocardial infarction (NSTEMI): cTn>99th ULN or CK-MB>99th ULN, along with one or more of the following conditions: persistent ischemic chest pain; ECG findings of new ST segment depression or T wave flattening, inversion; ECG showing abnormal segmental ventricular wall activity; and abnormal coronary angiography. (3) cTn negative, ischemic chest pain, ECG presented with transient ST segment depression or T wave low flat, inversion, and rare ST segment elevation (vasospasm angina pectoris).

### 1.3. Assessment of Coronary Angiography

All enrolled ACS patients underwent coronary angiography (CAG) with Philips Azurion, and then the degree of coronary narrowing was analyzed by two interventional physicians. According to the CAG results, the atherosclerosis of ACS was evaluated by two independent senior cardiologists applying the Gensini scoring system. ACS coronary artery stenosis degree and scoring of coronary artery lesion size were as followed: narrowing ≤ 25% contributed to 1 point, 26 ≤ narrowing ≤ 50% contributed to 2 points, 51 ≤ narrowing ≤ 75% contributed to 4 points, 76 ≤ narrowing ≤ 90% contributed to 8 points, 91 ≤ narrowing ≤ 99% contributed to 16 points, and complete occlusion contributed to 32 points. Next, each lesion score was multiplied by a factor that indicates the importance of the location of the lesion in the coronary circulation. The factor for the location of main left coronary artery was 5, the proximal segment of the anterior descending branch of the left coronary artery was 2.5, the proximal segment of the circumflex branch was 2.5, the middle segment of the anterior descending branch of the left coronary artery was 1.5, the right coronary artery was 1.0, the distal end of the anterior descending branch of the left coronary artery, the posterolateral artery and the obtuse artery, and the other segments were 0.5. At the end, Gensini score was calculated by the sum of each coronary segment score [11, 12]. The patients were categorized into three groups: 13 into the low (Gensini score < 25 points), 33 into intermediate (Gensini score 25-50 points), and 81 into high Gensini tertile (Gensini score ≥ 50 points) [13].

### 1.4. Laboratory Measurement and Index Calculation

Blood and plasma samples were taken immediately after admission. Cardiovascular biomarkers, including hydroxybutyrate dehydrogenase (HBDH), lactate dehydrogenase (LDH), creatine kinase (CK), creatine kinase myocardial band (CK-MB), cholesterol (CHOL), low-density lipoprotein cholesterol (LDL-C), triglyceride (TG) and creatinine (Cr), and vasculitis factors, including NO, endothelin-1 (ET-1), IL-8, and NO, were measured by the pathological laboratory of Shunde Hospital of Guangzhou University of Traditional Chinese Medicine. Plasma TMAO was measured using a double-antibody sandwich enzyme-linked immunosorbent test (ELISA, RX103049H, Ruixin Biotechnology, Quanzhou, China) and experiment was performed by Guangzhou Weijia Technology. Carotid IMT was measured by the Color Doppler (Philips epiq7c).

### 1.5. Statistical Analyses

Values of continuous variables were presented as medians with interquartile range (IQR). Kruskal-Wallis analysis of variance on ranks was used for comparisons of the three Gensini subgroups. Categorical variables were presented as numbers and percentages. The multivariate nonconditional logistic regression was used to analyze the correlation between Gensini score and vasculitis factors and cardiovascular biomarkers, the linear regression was used for the trend test. The Spearman correlation analysis was used between Gensini score and vasculitis factors and cardiovascular biomarkers levels in ACS patients. The multivariate logistic analysis was used to analyze the risk factors of atherosclerosis; the receiver operating curve (ROC) was used to analyze the value of TMAO in the prediction of ACS [14]. Statistical Package for the Social sciences (SPSS) 25.0 was used for all the analyses. A *p* value of <0.05 was considered statistically significant.

## 2. Results

### 2.1. Baseline Data

A total of 127 patients with ACS who underwent PCI, were divided into three groups according to Gensini score tertiles: low score tertile (Gensini score < 25 points; *n* = 13), intermediate score tertile (Gensini score 25-20 points; *n* = 33), and high score tertile (Gensini score > 50 points; *n* = 81). As shown in Table 1, no obvious association was observed between Gensini score and age, BMI, BNP, TNI, CK, CK-MB, HDBH, LDH, CHOL, LDL-C, TG, Cr, NO, ET-1, IL-8, and IMT (all *p* > 0.05). The plasma TMAO concentrations of patients with severe coronary atherosclerosis were higher than that of patients with moderate coronary atherosclerosis, and the plasma TMAO concentrations of patients with moderate coronary atherosclerosis were higher than that of patients with mild coronary atherosclerosis, the difference was statistically significant [4.73 (3.13, 4.62) versus 1.13 (0.63, 3.34) versus 0.79 (0.20, 1.29), *p* < 0.001].

### 2.2. Clinical Outcomes

**Figure 1:**
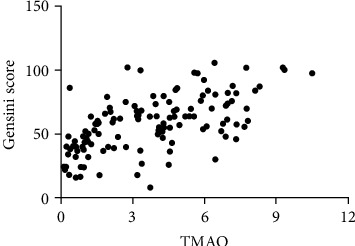
The relationship between TMAO and atherosclerosis.

**Figure 2:**
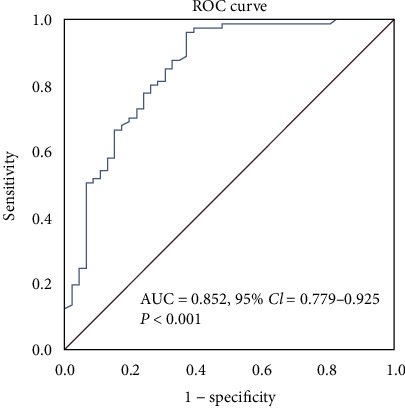
Receiver operating characteristics analysis. ROC curve was used to evaluate the discriminative capacity of TMAO for severe atherosclerosis.

Plasma TMAO concentrations were positively associated with Gensini score (OR = 0.629, *p* < 0.001) and Gensini integral (OR = 0.604, *p* < 0.001), as shown in Table 2 and Figure 1. Plasma TMAO significantly identified the severe atherosclerosis (*p* < 0.001). The AUC of TMAO for severe atherosclerosis was 0.852 (95%CI = 0.779 − 0.925). When the cut-off value of TMAO was set at 1.2715 pg/ml, the sensitivity and specificity of TMAO for identifying severe atherosclerosis are 96.3% and 63.0%, as shown in Figure 2. Linear regression analysis was used to analyze the relationship between risk factors and Gensini score in ACS patients, and we found TMAO is a risk factor for the severity of atherosclerosis [*β* = 3.358 (1.803, 4.914), *p* < 0.001], as shown in Table 3. In addition, after adjustment of potential confounding factors such as gender, diabetes, and BMI, LDL-C and plasma TMAO can explain 44.6% of the variation of atherosclerosis score, as shown in Table 3. Furthermore, logistic regression analysis showed plasma TMAO concentrations were positively associated with the severity of atherosclerosis (OR = 1.934, 95%CI = 1.522 − 2.459, *p* < 0.001), as shown in Table 4. TMAO was negatively correlated with age (OR = −0.224, *p* < 0.05), BNP (OR = −0.175, *p* < 0.05), IL-8 (OR = −0.324, *p* < 0.001), and positively correlated with NO (OR = 0.234, *p* < 0.01), as shown in Table 2. However, no obvious association was observed between Gensini score and age, BMI, plasma BNP, TNI, CK, CK-MB, HDBH, LDH, CHOL, LDL-C, TG, Cr, NO, ET-1, IL-8, and IMT index.

## 3. Discussion

Gensini score is the most used quantitative analysis of coronary artery disease, as it fully considers the number, location, and degree of coronary artery disease, which is a relatively scientific evaluation standard [15]. In present study, through performing Gensini score on ACS patients who underwent PCI, we found that plasma TMAO was positively correlated with Gensini score, which can act as a risk factor for severe atherosclerosis.

Yu et al., found that the plasma concentrations of TMAO, choline, creatinine, and carnitine in patients with coronary artery disease were significantly higher than those in patients with normal coronary arteries [16]. A case-control study in China found that high plasma TMAO concentrations were positively correlated with an increased risk of CHD, with or without adjusting for the main risk factors of CHD [17]. In addition, TMAO can increase the release of various inflammatory factors and increase the risk of atherosclerosis [18]. Some studies have found that TMAO can promote the formation and development of atherosclerosis, revealing that the concentration of plasma TMAO was closely related to cardiovascular risk [19, 20]. The higher plasma TMAO concentration, the more severe the atherosclerotic plaque in patients, and the higher the proportion of vulnerable plaque [21]. In our study, plasma TMAO concentration was positively correlated with Gensini score (OR = 0.629, *p* < 0.001) and Gensini integral (O = 0.604, *p* < 0.001), so it was associated with atherosclerosis. To further explore the discriminative capacity of TMAO for severe atherosclerosis, ROC analysis was performed. We found that TMAO could significantly identify the severe atherosclerosis (*p* < 0.001). The AUC of TMAO for severe atherosclerosis was 0.852 (95%CI = 0.779 − 0.925). The sensitivity and specificity of TMAO for identifying severe atherosclerosis were 96.3% and 63.0% when the cut-off value of TMAO was set at 1.2715 pg/ml.

Tan et al. conducted a prospective cohort study on 211 patients with STEMI using optical coherence tomography to evaluate the plaque morphology. Their results showed that the plasma TMAO concentration of patients with ST segment elevation myocardial infarction caused by plaque rupture was significantly higher than that of patients caused by plaque erosion. After adjustment of risk factors, Tan et al. reported that elevated plasma TMAO concentration was an independent predictor of plaque rupture [22]. A study found that in patients undergoing coronary artery stent implantation, the plasma TMAO concentration of patients with new atherosclerotic plaque was significantly higher than that of patients without early atherosclerotic plaque. It was also found that in patients with coronary plaque formation, the plasma TMAO concentration in patients with plaque rupture was higher than that in patients without plaque rupture. It is suggested that plasma TMAO concentration can be used as a reliable risk factor of ACS and predictor of plaque instability [23]. One study showed that TMAO could independently predict the occurrence of short-term (6 months) and long-term (2 years) all-cause mortality or adverse outcomes of recurrent myocardial infarction after AMI [24]. Other studies have shown that TMAO can be used as a prognostic indicator of short-term and long-term cardiovascular events in acute coronary syndrome [25, 26]. In our study, logistic regression analysis showed plasma TMAO concentrations were positively associated with the severity of atherosclerosis in coronary artery (OR = 1.934, 95%CI = 1.522 − 2.459, *p* < 0.001), indicating that TMAO can be a risk factor for ACS.

It has been reported that TMAO induced inflammation in human umbilical vein endothelial cells and artery of *Apoe* gene knockout (*Apoe^–/–^*) mice by enhancing the activity of caspase-1 and mitochondrial oxidative stress, which then led to atherosclerosis [27]. TMAO has also been reported to promote the aggregation of activated leukocyte to endothelial cell. Compared with the control group, the expression of inflammatory genes was increased in LDL receptor deficient mice fed choline diet [28]. TMAO induced endothelial dysfunction by destroying endothelial connexin, enhancing vascular inflammation and oxidative stress, and atherosclerosis [29]. Wu et al. found that treatment of TMAO damaged the structure and function of mitochondria in *Apoe^–/–^* mice via elevating the expression of succinate dehydrogenase complex B subunit, increasing the production of reactive oxygen species, promoting endothelial cell apoptosis, and enhancing the release of proinflammatory cytokines, thereby exacerbating atherosclerosis [30]. In our study, we found that TMAO negatively correlated with age, IL-8, and BNP and positively correlated with NO. NO is considered to be the most effective endogenous vasodilator *in vivo* [31], and it also plays a role in inhibiting platelet aggregation, activating inflammation, oxidative stress, promoting the migration and proliferation of vascular smooth muscle cells, and enhancing leukocyte adhesion by maintaining the dynamic balance in the vascular wall [32], thus delaying atherosclerosis. This study suggested that TMAO is negatively correlated with NO, therefore TMAO may cause atherosclerosis by reducing NO. IL-8 can stimulate the proliferation of vascular smooth muscle cells and accelerate the formation of atherosclerotic plaque [33, 34]. In our study, we could not find atherosclerosis were related to IL-8, which may be due to relatively small sample size. Therefore, it cannot be determined whether TMAO causes atherosclerosis through IL-8. BNP, the main cardiovascular biomarker, rises with the increase of ventricular load pressure and has been used to evaluate cardiac function. It can antagonize renin angiotensin aldosterone receptor, reduce blood pressure, induce natriuresis/diuresis, and act as a potent vasodilator, thus slowing down heart failure and protecting cardiac function. In this study, we observed that plasma TMAO negatively correlated with BNP, which indicates that TMAO may aggravate heart failure by reducing BNP release. Unfortunately, in our current study of patients with ACS, no statistical correlation was found between plasma inflammatory factors, blood lipids, and atherosclerosis, therefore it cannot be concluded that TMAO increases the risk of atherosclerosis through inflammation or dyslipidemia.

This study has some limitations which will need to be addressed in future research. Firstly, this cross-sectional study did not determine whether there is a causal relationship between plasma TMAO and ACS. Secondly, data of this study came from a single center, which did not rule out the influence of regional differences. In addition, the plasma TMAO concentration was only measured in one time point, which could not reflect the impact of its dynamic changes on the pathogenesis and prognosis of ACS; moreover, the ACS population in our study was not statistically correlated with IMT, ET-1, and blood lipids, which may be due to the relative small size of the cohort. To determine whether the plasma TMAO concentration is related to ACS, and whether the plasma TMAO concentration is related to the degree of coronary atherosclerosis, it is necessary to carry out a prospective study with a larger cohort of patients in the future study.

## 4. Conclusion

Overall, the present study suggests plasma TMAO concentrations positively associated with atherosclerosis in coronary artery of ACS and TMAO can act as a risk factor for severe atherosclerosis. Plasma TMAO may promote the progression of atherosclerosis and lead to ACS. Therefore, lowering the concentrations of plasma TMAO may provide protections to against atherosclerosis and the related diseases, thereby reducing the incidence rate of ACS.

## Figures and Tables

**Table 1:** Baseline characteristics of patients.

Metric	Mild (*n* = 13)	Moderate (*n* = 33)	Severe (*n* = 81)	*p*
Age, M (P25, P75)	59 (54, 67)	64 (55, 71)	60 (52, 70)	0.669
Gender (male), *n* (%)	9 (69.2)	26 (78.8)	57 (70.4)	0.635
Smoking, *n* (%)	7 (53.8)	19 (57.6)	46 (56.8)	0.974
Hypertension, *n* (%)	7 (53.8)	25 (75.8)	44 (54.3)	0.095
Diabetes mellitus, *n* (%)	3 (23.1)	13 (39.4)	24 (29.6)	0.470
ACS				0.577
STEMI, *n* (%)	6 (46.2)	14 (42.4)	38 (46.9)	
NSTEMI, *n* (%)	1 (7.7)	6 (18.2)	19 (23.5)	
UA, *n* (%)	6 (46.2)	13 (39.4)	24 (29.6)	
The ST changes of the ECG were observed				0.377
No change, *n* (%)	6 (46.2)	10 (30.3)	19 (23.5)	
Elevation, *n* (%)	6 (46.2)	14 (42.4)	40 (49.4)	
Shift down, *n* (%)	1 (7.7)	9 (27.3)	22 (27.2)	
Gensini score, M (P25, P75)	18 (16, 24)	40 (37, 45)	68 (59, 81)	<0.001
BMI, M (P25, P75)	26.1 (25.1, 26.4)	25.2 (24.7, 26.2)	24.8 (23.8, 26.4)	0.262
TNI, M (P25, P75)	0.32 (0.02, 13.03)	0.33 (0.02, 8.36)	0.20 (0.02, 5.23)	0.981
BNP, M (P25, P75)	139.80 (28.18, 207.62)	111.00 (81.61, 250.87)	84.04 (50.39, 200.65)	0.142
CK-MB, M (P25, P75)	18.32 (14.65, 42.60)	52.10 (19.00, 206.83)	29.50 (17.30, 91.75)	0.079
CK, M (P25, P75)	124.85 (98.08, 446.30)	376.60 (118.45, 1576.73)	204.50 (88.40, 844.60)	0.087
HBDH, M (P25, P75)	213.45 (143.63, 451.85)	268.90 (164.98, 688.20)	204.05 (146.63, 418.38)	0.333
LDH, M (P25, P75)	414.00 (203.60, 570.53)	398.40 (202.83, 957.53)	315.90 (198.95, 626.06)	0.793
CHOL, M (P25, P75)	4.85 (4.10, 5.55)	5.18 (4.08, 6.36)	5.03 (4.13, 5.81)	0.846
LDL-CD, M (P25, P75)	3.32 (2.54, 3.95)	3.70 (2.57, 4.41)	3.57 (2.69, 4.22)	0.832
TG, M (P25, P75)	1.35 (0.96, 2.02)	1.57 (1.01, 2.25)	1.41 (1.04, 2.00)	0.850
Cr, M (P25, P75)	83.10 (63.80, 100.30)	84.80 (69.00, 107.70)	79.20 (65.95, 96.75)	0.604
IMT, M (P25, P75)	11.70 (6.70, 16.25)	11.80 (8.65, 14.50)	10.00 (8.80, 14.10)	0.814
ET-1, M (P25, P75)	0.52 (0.37, 1.04)	0.40 (0.27, 0.60)	0.40 (0.28, 0.70)	0.262
IL-8, M (P25, P75)	4.78 (3.38, 9.47)	3.38 (1.54, 6.53)	3.38 (1.54, 7.29)	0.222
NO, M (P25, P75)	2.63 (1.18, 6.08)	2.02 (0.52, 3.78)	3.14 (1.23, 6.12)	0.071
TMAO, M (P25, P75)	0.79 (0.20, 1.29)	1.13 (0.63, 3.34)	4.73 (3.13, 6.42)	<0.001

**Table 2:** Analysis of correlated factors.

	The Gensini score is assigned to the score	Gensini grading	TMAO
Age	-0.009	-0.049	-0.224^∗^
BMI	0.018	-0.122	0.001
TNI	0.035	0.006	-0.129
BNP	-0.164	-0.115	-0.175^∗^
CK-MB	0.075	0.049	0.072
CK	0.002	-0.042	0.017
HBDH	0.001	-0.081	-0.060
LDH	0.045	-0.036	-0.013
CHOL	0.088	-0.006	-0.039
LDL-C	0.127	0.021	0.004
TG	-0.087	-0.001	0.002
Cr	-0.117	-0.061	-0.161
IMT	0.001	-0.054	-0.088
ET-1	-0.016	-0.097	-0.166
I L-8	-0.084	-0.098	-0.324^∗∗∗^
NO	0.162	0.154	0.234^∗∗^
TMAO	0.629^∗∗∗^	0.604^∗∗∗^	—

Spearman correlation analysis, ^∗^*p* < 0.05, ^∗∗^*p* < 0.01, and ^∗∗∗^*p* < 0.001.

**Table 3:** Linear regression analysis of the Gensini scores.

Argument	Single factor		Multiple factor^#^	
	*β* (95% CI)	*p*	*β* (95% CI)	*p*
Age	-0.039 (-0.381, 0.303)	0.822	NA	NA
Gender (male)	1.978 (-6.796, 10.752)	0.656	NA	NA
Smoke	1.045 (-6.871, 8.962)	0.794	NA	NA
Hypertension	-5.190 (-13.141, 2.760)	0.199	NA	NA
Diabetes mellitus	-2.048 (-10.487, 6.391)	0.632	NA	NA
ACS				
UA	Ref	Ref	NA	NA
STEMI	4.050 (-4.781, 12.880)	0.366	NA	NA
NSTEMI	9.422 (-1.479, 20.323)	0.090	NA	NA
The ST changes of the ECG were observed				
No change	Ref	Ref	NA	NA
Elevate	6.010 (-3.279, 15.298)	0.203	NA	NA
Shift down	10.940 (0.258, 21.621)	0.045	NA	NA
BMI	0.413 (-0.078, 0.903)	0.098	-0.933 (-1.422, -0.445)	<0.001
TNI	0.051 (-0.236, 0.339)	0.725	NA	NA
BNP	0.002 (-0.004,0.008)	0.491	NA	NA
CK-MB	0.024 (-0.017, 0.064)	0.250	NA	NA
CK	0.002 (-0.001, 0.006)	0.172	NA	NA
HBDH	-0.002 (-0.016, 0.011)	0.732	NA	NA
LDH	0.003 (-0.005, 0.012)	0.448	NA	NA
CHOL	1.899 (-1.09, 4.888)	0.211	NA	NA
LDL-C	3.203 (-0.149, 6.556)	0.061	NA	NA
TG	-0.759 (-4.427, 2.909)	0.683	NA	NA
Cr	0.030 (-0.030, 0.090)	0.329	NA	NA
IMT	0.112 (-0.447, 0.670)	0.693	NA	NA
ET-1	1.548 (-4.845, 7.940)	0.633	NA	NA
I L-8	-0.425 (-1.103, 0.253)	0.218	NA	NA
NO	0.266 (-0.255, 0.787)	0.315	NA	NA
TMAO	5.375 (4.156, 6.594)	<0.001	3.358 (1.803, 4.914)	<0.001

^#^Using a stepwise multifactor linear regression analysis, the included factors with a univariate *p* < 0.200 were entered into the multivariate analysis.

**Table 4:** The logistic regression analysis of the factors associated with severe atherosclerosis.

Argument	Single factor		Multiple factor^#^	
	OR (95% CI)	*p*	OR (95% CI)	*p*
Age	0.990 (0.959, 1.022)	0.538	NA	NA
Gender (male)	1.340 (0.585, 3.068)	0.489		
Smoke	0.989 (0.477, 2.053)	0.977	NA	NA
Hypertension	0.520 (0.242, 1.118)	0.094	NA	NA
Diabetes mellitus	0.789 (0.365, 1.708)	0.548	NA	NA
ACS				
UA	Ref	Ref	NA	NA
STEMI	1.504 (0.669, 3.380)	0.323	4.528 (1.087, 18.861)	0.038
NSTEMI	2.149 (0.748, 6.172)	0.155	4.277 (1.076, 16.996)	0.039
The ST changes of the ECG were observed				
No change	Ref	Ref	NA	NA
Elevate	0.540 (0.198, 1.468)	0.227	NA	NA
Shift down	0.909 (0.362, 2.282)	0.839	NA	NA
BMI	1.014 (0.966, 1.064)	0.575	NA	NA
TNI	0.996 (0.970, 1.022)	0.746	NA	NA
BNP	1.000 (1.000, 1.001)	0.360	NA	NA
CK-MB	0.999 (0.996, 1.003)	0.788	NA	NA
CK	1.0000 (1.000, 1.000)	0.918	NA	NA
HBDH	0.999 (0.998, 1.000)	0.112	0.998 (0996, 1.000)	0.048
LDH	1.000 (0.999, 1.001)	0.912	NA	NA
CHOL	1.001 (0.759, 1.322)	0.992	NA	NA
LDL-CD	.105 1 (0.802, 1.522)	0.541	NA	NA
TG	1.030 (0.731, 1.450)	0.867	NA	NA
Cr	1.000 (0.994, 1.005)	0.896		
IMT	0.989 (0.940, 1.041)	0.672	NA	NA
ET-1	0.970 (0.540, 1.744)	0.920	NA	NA
IL-8	0.969 (0.911, 1.031)	0.320	NA	NA
NO	.028 1 (0.967, 1.093)	0.375	NA	NA
TMAO	1.934 (1.522, 2.459)	<0.001	1.952 (1.491, 2.557)	<0.001

## Data Availability

The original contributions presented in the study will be made available by the authors, without undue reservation.

## References

[1] Wang Y., Yong Q., Liu X., Zhang L., Bao J. J. (2019). Coronary artery lesion and carotid atherosclerosis study on the correlation between vulnerability of sclerotic plaque. *Chinese Journal of Ultrasound Medicine*.

[2] Bu J., Chen Z. W., Cui X. T. (2020). Metabolic abnormalities and prevention and treatment of cardiovascular diseases in Chinese adults. *Shanghai Medical Journal*.

[3] Vos T., Abajobir A. A., Abate K. H. (2017). Global, regional, and national incidence, prevalence, and years lived with disability for 328 diseases and injuries for 195 countries, 1990-2016: a systematic analysis for the global burden of disease study 2016. *The Lancet*.

[4] Tomaniak M., Chichareon P., Onuma Y. (2019). Benefit and risks of aspirin in addition to ticagrelor in acute coronary syndromes. *JAMA Cardiology*.

[5] Wang Z., Zhao Y. (2018). Gut microbiota derived metabolites in cardiovascular health and disease. *Protein & Cell*.

[6] Tang W. H., Wang Z., Levison B. S. (2013). Intestinal microbial metabolism of phosphatidylcholine and cardiovascular risk. *The New England Journal of Medicine*.

[7] Janeiro M. H., Ramírez M. J., Milagro F. I., Martínez J., Solas M. (2018). Implication of Trimethylamine N-oxide (TMAO) in disease: potential biomarker or new Therapeutic. *Target Nutrients*.

[8] Tang W. H. W., Li D. Y., Hazen S. L. (2019). Dietary metabolism, the gut microbiome, and heart failure. *Nature Reviews. Cardiology*.

[9] Geng J., Yang C., Wang B. (2018). Trimethylamine N-oxide promotes atherosclerosis via CD36-dependent MAPK/JNK pathway. *Biomedicine & Pharmacotherapy*.

[10] Sun X., Jiao X., Ma Y. (2016). Trimethylamine N-oxide induces inflammation and endothelial dysfunction in human umbilical vein endothelial cells via activating ROS-TXNIP-NLRP3 inflammasome. *Biochemical and Biophysical Research Communications*.

[11] Gensini G. G. (1983). A more meaningful scoring system for determining the severity of coronary heart disease. *The American Journal of Cardiology*.

[12] Wang K. Y., Zheng Y. Y., Wu T. T., Ma Y. T., Xie X. (2022). Predictive value of Gensini score in the long-term outcomes of patients with coronary artery disease who underwent PCI. *Frontiers in Cardiovascular Medicine*.

[13] Xia M. W., Shao Z. B., Liang G. Q. (2019). Correlation of platelet parameters and serum IL-6 levels with severity of coronary lesions in coronary heart disease. *Chinese Journal of Evidence-Based Medicine*.

[14] Cao R. Y., Yang J., Zheng Y. (2021). The potential value of copeptin and pentraxin3 for evaluating the severity of coronary stenosis in patients with coronary artery disease. *Clinical Biochemistry*.

[15] Huo Y., Fang W. Y. (2019). *Training Materials for Interventional Therapy of Coronary Heart Disease, 2018 ed*.

[16] Yu D., Shu X. O., Rivera P. E. S. (2019). Urinary levels of trimethylamine-N-oxide and incident coronary heart disease: a prospective investigation among urban Chinese adults. *Journal of the American Heart Association*.

[17] Zhong Z., Liu J., Zhang Q. (2019). Targeted metabolomic analysis of plasma metabolites in patients with coronary heart disease in southern China. *Medicine (Baltimore)*.

[18] Ma G. H., Pan B., Chen Y. (2017). Trimethylamine N-oxide in atherogenesis: impairing endothelial self-repair capacity and enhancing monocyte adhesion. *Bioscience Reports*.

[19] Guasti L., Galliazzo S., Molaro M. (2021). TMAO as a biomarker of cardiovascular events: a systematic review and meta-analysis. *Internal and Emergency Medicine*.

[20] Li J., Tan Y., Zhou P. (2021). Association of trimethylamine N-oxide levels and calcification in culprit lesion segments in patients with ST-segment elevation myocardial infarction evaluated by optical coherence tomography. *Frontiers in Cardiovascular Medicine*.

[21] Thomas M. S., Fernandez M. L. (2021). Trimethylamine N – oxide (TMAO), diet and cardiovascular disease. *Current Atherosclerosis Reports*.

[22] Tan Y., Sheng Z., Zhou P. (2019). Plasma trimethylamine N-oxide as a novel biomarker for plaque rupture in patients with ST-segment-elevation myocardial infarction. *Circulation: Cardiovascular Interventions*.

[23] Zuo K., Li J., Li K. (2019). Disordered gut microbiota and alterations in metabolic patterns are associated with atrial fibrillation. *Gigascience*.

[24] Suzuki T., Heaney L. M., Jones D. J., Ng L. L. (2017). Trimethylamine N-oxide and risk stratification after acute myocardial infarction. *Clinical Chemistry*.

[25] Li X. S., Obeid S., Klingenberg R. (2017). Gut microbiota-dependent trimethylamine N-oxide in acute coronary syndromes: a prognostic marker for incident cardiovascular events beyond traditional risk factors. *European Heart Journal*.

[26] Haghikia A., Li X. S., Liman T. G. (2018). Gut microbiota-dependent trimethylamine N-oxide predicts risk of cardiovascular events in patients with stroke and is related to proinflammatory monocytes. *Arteriosclerosis, Thrombosis, and Vascular Biology*.

[27] Chen M. L., Zhu X. H., Ran L., Lang H. D., Yi L., Mi M. T. (2017). Trimethylamine-N-oxide induces vascular inflammation by activating the NLRP3 inflammasome through the SIRT3-SOD2-mtROS signaling pathway. *Journal of the American Heart Association*.

[28] Seldin M. M., Meng Y., Qi H. (2016). Trimethylamine N-oxide promotes vascular inflammation through signaling of mitogen-activated protein kinase and nuclear Factor‐*κ*B. *Journal of the American Heart Association*.

[29] Singh G. B., Zhang Y., Km B., Koka S. (2019). High mobility group box 1 mediates TMAO-induced endothelial dysfunction. *International Journal of Molecular Sciences*.

[30] Wu P., Chen J., Chen J. (2020). Trimethylamine N-oxide promotes apoE-/- mice atherosclerosis by inducing vascular endothelial cell pyroptosis via the SDHB/ROS pathway. *Journal of Cellular Physiology*.

[31] Mudau M., Genis A., Lochner A., Strijdom H. (2012). Endothelial dysfunction: the early predictor of atherosclerosis. *Cardiovascular Journal of Africa*.

[32] Tousoulis D., Simopoulou C., Papageorgiou N. (2014). Endothelial dysfunction in conduit arteries and in microcirculation novel therapeutic approaches. *Pharmacology & Therapeutics*.

[33] Biondi-Zoccai G., Garmendia C. M., Abbate A. (2020). Atherothrombosis prevention and treatment with anti-interleukin-1 agents. *Current Atherosclerosis Reports*.

[34] Zheng Z. H., Zeng X., Nie X. Y. (2019). Retracted: interleukin-1 blockade treatment decreasing cardiovascular risk. *Clinical Cardiology*.

